# Opicapone for the treatment of early wearing-off in levodopa-treated Parkinson’s disease: pooled analysis of patient level data from two randomized open-label studies

**DOI:** 10.1007/s00415-024-12614-8

**Published:** 2024-08-21

**Authors:** Joaquim J. Ferreira, Jee-Young Lee, Hyeo-il Ma, Beomseok Jeon, Werner Poewe, Angelo Antonini, Fabrizio Stocchi, Daniela M. Rodrigues, Miguel M. Fonseca, Guillermo Castilla-Fernández, Joerg Holenz, José-Francisco Rocha, Olivier Rascol, on behalf of the ADOPTION study investigators

**Affiliations:** 1 IMM - Instituto de Medicina Molecular João Lobo Antunes, Faculdade de Medicina, Universidade de Lisboa, Lisbon, Portugal; 2 CNS – Campus Neurológico, Torres Vedras, Portugal; 3 Department of Neurology, SMG-SNU Boramae Medical Center, Seoul, Korea; 4 Department of Neurology, Hallym University Sacred Heart Hospital, Anyang, Korea; 5 Department of Neurology, Seoul National University Hospital, Seoul, Korea; 6 Department of Neurology, Medical University of Innsbruck, Innsbruck, Austria; 7 Department of Neurosciences, University of Padova, Padua, Italy; 8 University San Raffaele Roma and Institute for Research and Medical Care IRCCS San Raffaele, Rome, Italy; 9 BIAL – Portela and Ca S.A., Coronado, Portugal; 10 BIAL - R&D Investments, S.A., Coronado, Portugal; 11 Clinical Investigation Center CIC1436, Departments of Neurosciences and Clinical Pharmacology and NS-Park/FCRIN Network, University of Toulouse 3, University Hospital of Toulouse, INSERM, Toulouse, France

**Keywords:** COMT, Levodopa, Motor fluctuations, Opicapone, Parkinson’s disease, Wearing-off

## Abstract

**Background:**

The wearing-off phenomenon is a key driver of medication change for patients with Parkinson’s disease (PD) treated with levodopa. Common first-line options include increasing the levodopa dose or adding a catechol-O-methyltransferase (COMT) inhibitor, but there are no trials comparing the efficacy of these approaches. We evaluated the effectiveness of adjunct opicapone versus an additional 100 mg levodopa dose in PD patients with early wearing-off using pooled data from 2 randomized studies.

**Methods:**

The ADOPTION study program included two similarly designed 4-week, open-label studies conducted in South Korea (NCT04821687) and Europe (NCT04990284). Patients with PD, treated with 3–4 daily doses of levodopa therapy and with signs of early wearing-off were randomized (1:1) to adjunct opicapone 50 mg or an additional dose of levodopa 100 mg. Patient-level data from the two studies were pooled.

**Results:**

The adjusted mean [SE] change from baseline to week 4 in absolute OFF time (key endpoint) was − 62.8 min [8.8] in the opicapone group and − 33.8 min [9.0] in the levodopa 100 mg group, the difference significantly favoring opicapone (− 29.0 [− 53.8, − 4.2] min, *p* = 0.02). Significant differences in the Movement Disorder Society—Unified Parkinson’s Disease Rating Scale Part III subscore (− 4.1 with opicapone vs − 2.5 with levodopa 100 mg), also favored opicapone (− 1.7 [− 3.3, − 0.04], *p* < 0.05). Dyskinesia was the most frequently reported adverse event (opicapone 7.2% vs. levodopa 100 mg 4.2%).

**Conclusions:**

In these short-term trials, introducing adjunct opicapone was more effective at reducing OFF time than adding another 100 mg levodopa dose in PD patients with early signs of wearing-off.

**Supplementary Information:**

The online version contains supplementary material available at 10.1007/s00415-024-12614-8.

## Introduction

In the last decade, there has been a general return to the use of levodopa as initial monotherapy for most patients with Parkinson’s disease (PD) [1], but with the aim of keeping the dose as low as possible for as long as possible to avoid the development of levodopa motor complications which are a significant source of disability. Often, one of the first motor complications to emerge are ‘wearing-off’ motor fluctuations reflecting a decrease in the duration of effect of each individual dose of levodopa with increasing disease progression and duration of drug treatment [2]. Wearing-off fluctuations can already emerge within the first few years after levodopa initiation [3], but early wearing-off may go unrecognized at office visits, particularly in patients with a shorter disease duration [4]. Predictors of wearing-off (in addition to duration of treatment and disease severity) include a younger age, weight, and female sex [4, 5], with women showing an 80% increased risk for wearing-off [6].

The development of wearing-off fluctuations is a key driver of medication changes [7]. One of the most common approaches to treating early wearing-off is to alter the levodopa dosing regimen, for example, by shortening interdose intervals and/or adding another dose. However, the introduction of a 4th or 5th levodopa dose poses challenges for adherence [8, 9] and is often only effective for a limited period after which further increases in dosing frequency usually are necessary (in turn increasing the risk of inducing dyskinesias). COMT inhibitors, such as opicapone, are recommended by evidence-based guidelines as effective treatment for patient with motor complications [10]. Such recommendations are based on a wealth of data from clinical trials such as the BIPARK pivotal studies [11, 12]. However, these studies enrolled patients who had well established motor fluctuations (average disease duration of 8 years and time with motor fluctuations of 3 years) and a mean daily OFF time of over 6 h [13] indicative of a population with chronic motor complications. However, *post-hoc* analyses of the pivotal studies suggested the efficacy of opicapone 50 mg in those patients who had developed motor fluctuations within the prior 2 years (placebo-adjusted reduction of OFF time of  − 68.5 min, *p* = 0.0003 vs. placebo) [13, 14].

The e**A**rly levo**D**opa with **O**picapone in **P**arkinson’s pa**T**ients w**I**th mot**O**r fluctuatio**N**s (ADOPTION) clinical program aimed to evaluate the efficacy and safety of opicapone 50 mg in PD patients with early wearing-off. To improve study feasibility, the overall Phase 4 program has been designed such that all component studies (at national or regional levels) can be kept relatively small but with the intention of pooling for additional power and to support international generalizability. Thus far, two similarly designed trials have been conducted in Europe and South Korea. The results of the South Korean study have recently been published [15]. Here, we report findings from the pooled analysis of both studies.

## Methods

### Study conduct

The ADOPTION studies were two randomized, parallel-group, multicenter, prospective, open-label, exploratory, Phase 4 studies conducted from June 2021 to August 2022 in Korea and from November 2021 to April 2023 in Europe (Germany, Italy, Portugal, Spain, and the UK). Both studies were conducted in accordance with the Declaration of Helsinki; each study protocol was approved by the ethics committee at each site and all patients provided informed consent.

### Study population

Full protocol details have been previously described [15]. Briefly, male or female patients aged ≥ 30 years were eligible if they had a diagnosis of PD, a Hoehn and Yahr stage of 1–3 (during ON) and were being treated with 3–4 daily doses of levodopa/dopa-decarboxylase inhibitor therapy (maximum 600 mg) for ≥ 4 weeks prior to study entry. Patients had early signs of wearing-off, defined as having at least 1 h of daily OFF time for at least 4 weeks, but for less than 2 years. Key exclusion criteria included atypical parkinsonism, severe and/or unpredictable OFF periods, or awake daily OFF time of more than 5 h. Previous or planned surgery for deep brain stimulation and treatment with monoamine oxidase inhibitors (except for selegiline ≤ 10 mg/day oral, ≤ 1.25 mg/day buccal absorption formulations, rasagiline ≤ 1 mg/day, and safinamide ≤ 100 mg/day), opicapone, entacapone, tolcapone and anti-emetics with anti-dopaminergic action (except domperidone) were prohibited during the study (withdrawn ≥ 4 weeks before screening).

### Study design

Following screening, eligible patients were randomized (1:1) to opicapone 50 mg or levodopa 100 mg for 4 weeks in addition to their current levodopa therapy. Opicapone was administered once daily at bedtime 1 h after levodopa intake, and levodopa 100 mg was either taken as one full additional administration or by increasing one or more doses of the established levodopa regimen without altering dose frequency. During the first 2 weeks of study treatment, the total daily dose of levodopa (excluding the additional 100 mg levodopa dose) or the intake intervals could be adjusted in case of dopaminergic adverse events (AEs). However, daily doses were kept stable for the last 2 weeks of the study.

### Study endpoints

The key efficacy endpoint was the change from baseline to the end of study treatment in absolute OFF time, as assessed by daily paper patient diaries (mean of the 3 preceding diary days before each visit) [16]. We also assessed the change from baseline to the end of study treatment in the proportion of patients (i.e., responders) achieving (i) ≥ 1 h reduction in absolute OFF time and (ii) ≥ 1 h increase in absolute total ON time. Other diary-based efficacy variables were changes from baseline in the percentage of OFF time, in absolute ON time, in the percentage of ON time, and in ON time with and without dyskinesia. Percentages of OFF and ON time were calculated as the sum in minutes from 30-min periods classified as OFF or ON state divided by the total time awake.

Scale-based efficacy variables were the change from baseline in the Movement Disorder Society-Sponsored Revision of the Unified Parkinson’s Disease Rating Scale (MDS-UPDRS) [17, 18] assessed in the ON state, 8-item Parkinson’s Disease Questionnaire (PDQ-8) [19], and the Clinical Global Impression of Improvement (CGI-I) and Patient Global Impression of Improvement (PGI-I). Levodopa equivalent daily dose (LEDD) was calculated for monoamine oxidase B (MAO-B) inhibitors and dopamine agonists using the existing conversion factors [20].

Safety was assessed for the duration of the study and up to 2 weeks after the end of study by evaluating AEs, serious AEs, drug-related AEs, and AEs leading to discontinuation.

### Statistical analyses

Similarities in the design of the two clinical trials permitted a pooled analysis. This pooled analysis was based on integration of individual participant data. Like the two component studies, this preplanned integrated analysis was designed to be exploratory—without hierarchical hypothesis-testing (due to lack of previous trial data on the expected magnitude of effect for levodopa 100 mg) [15]. Patient-level data were combined and analyzed. ANCOVA models were used to evaluate the applicable endpoints as independent variables, treatment arm as fixed effect and baseline values as covariate. Only patients with available (non-missing) data for a particular variable were included in the calculation of a percentage. A mixed-effect linear model with repeated measures (MMRM) were used to evaluate the applicable endpoints as independent variables, with fixed effect for treatment group, visit, interaction between treatment group and visit, and a covariance for the baseline value (visit and corresponding interaction was not included for secondary efficacy endpoints). Responder rates of change in OFF/ON time were compared between the treatment groups using the Chi-square test.

Population sets were defined as the safety set, which included all randomly assigned patients who received at least one dose of study drug and the full analysis set (FAS), which included all randomly assigned patients who took at least one dose of study drug and had at least one efficacy assessment after baseline. Only patients with available (non-missing) data for a particular variable were included in the calculation of a percentage.

## Results

### Study population

Of the 244 patients enrolled across both studies, 243 patients were randomized and treated with either opicapone 50 mg (N = 125) or levodopa 100 mg (N = 118). Overall, 225 (92%) patients completed the studies; 18 patients (opicapone n = 10, levodopa n = 8) early discontinued (Fig. e1).

Baseline demographics and disease characteristics were similar between the two treatment arms (Table 1) and were consistent with an early fluctuating population. Data for the 2 separate studies are given in Table e1. Overall, the average duration of PD was 5.2 years, and the mean baseline levodopa daily dose was ~ 405 mg/day, distributed mostly over three (80%) daily intakes. Patients had a mean (SD) daily OFF time of 3.4 (1.0) h (~ 22% of their awake time) and most ON time was without dyskinesia (~ 89% of total ON time). Concomitant PD medication was common, with 62% of patients already receiving a dopamine agonist and 54% receiving an MAO-B inhibitor; the mean LEDD being 566 mg/day.

**Table 1:** Baseline characteristics (randomized set)

	Opicapone 50 mg N = 126	Levodopa 100 mg N = 118	Total N = 244
Age, year	64.1 (8.3)	64.6 (9.1)	64.3 (8.7)
Male, n (%)	65 (51.6)	63 (52.9)	128 (52.8)
Hoehn and Yahr, stage	2.0 (0.5)	2.1 (0.5)	2.0 (0.5)
PD duration, year	5.1 (3.6)	5.3 (3.6)	5.2 (3.6)
MDS-UPDRS motor score	23.7 (10.8)	24.7 (11.5)	24.2 (11.1)
PDQ-8 summary index	17.4 (13.2)	18.4 (14.6)	17.9 (13.9)
Daily OFF time, h	3.4 (1.0)	3.4 (1.0)	3.4 (1.0)
Total ON time, h	12.8 (1.6)	12.8 (1.6)	12.8 (1.6)
ON time without dyskinesia, h	11.6 (2.6)	11.2 (3.3)	11.4 (3.0)
Levodopa dose at baseline, mg	398.3 (117.4)	412.4 (119.5)	405.2 (118.7)
Patients receiving 3 or 4 levodopa intakes per day, n (%)^a^			
* 3 intakes*	101 (80.2)	93 (78.2)	194 (79.5)
* 4 intakes*	24 (19.0)	25 (21.0)	49 (20.1)
Concomitant therapy, n (%)^a^	106 (84.1)	99 (83.9)	205 (84.0)
Dopamine agonist	77 (61.1)	75 (63.6)	152 (62.3)
Pramipexole	56 (44.4)	53 (44.9)	109 (44.7)
Rotigotine	5 (4.0)	5 (4.2)	10 (4.1)
Ropinirole	22 (17.5)	19 (16.1)	41 (16.8)
MAO-B inhibitor	67 (53.2)	67 (56.8)	134 (54.9)
Rasagiline	59 (46.8)	51 (43.2)	110 (45.1)
Safinamide	7 (5.6)	13 (11.0)	20 (8.2)
Selegiline	2 (1.6)	3 (2.5)	5 (2.0)
LEDD^b^, mg	556.8 (166.5)	575.6 (170.7)	565.9 (168.5)

Data are mean (SD) unless otherwise specified

^a^One patient took 5 intakes in the opicapone 50 mg group; ^b^calculated for Levodopa, MAO-Bi and dopamine agonists only

*LEDD* levodopa equivalent daily dose, *MAO-Bi Monoamine oxidase B inhibitor; MDS-UPDRS* Movement Disorder Society-sponsored revision of the Unified Parkinson’s Disease Rating Scale, *PD* Parkinson’s disease, *PDQ* Parkinson’s disease questionnaire

### Efficacy

**Fig. 1:**
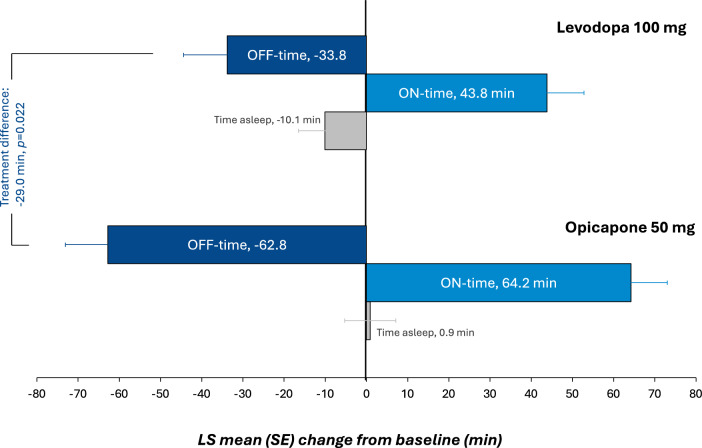
LS mean (SE) change from baseline to end of study treatment in absolute OFF, ON and asleep time. The treatment difference for change in ON time was non-significant. *LS* least square, *SE* standard error

Results for the integrated efficacy analysis are summarized in Table 2. For the key efficacy endpoint, the adjusted (least squares [LS]) mean (SE) change from baseline to end of study treatment in absolute OFF time was − 62.8 min [8.8] in the opicapone 50 mg and − 33.8 min [9.0] in the levodopa 100 mg group, resulting in a significant difference of − 29.0 [− 53.8, − 4.2] min favoring opicapone 50 mg (*p* = 0.022) (Fig. 1). Results were similar in males and females (Table e2). The percentage of OFF time reduction was also significantly higher with opicapone vs levodopa (*p* = 0.03), and the proportion of patients with a reduction in OFF time of at least 1 h was numerically higher in the opicapone 50 mg group than in the levodopa 100 mg group (56.7% vs. 45.6%, respectively).

**Table 2:** Change from baseline to week 4 in outcome measures (full analysis set)

	Opicapone 50 mg N = 122	Levodopa 100 mg N = 118
OFF time (min)		
LS mean (SE) change from baseline	− 62.8 (8.8)	− 33.8 (9.0)
LS mean difference versus levodopa 100 mg (95% CI)	− 29.0 (− 53.8, − 4.2)	
* p* value for opicapone 50 mg versus levodopa 100 mg	**0.0222**	
Percent OFF time (%)		
LS mean (SE) change from baseline	− 6.5 (0.9)	− 3.7 (0.9)
LS mean difference versus levodopa 100 mg (95% CI)	− 2.8 (− 5.4, − 0.3)	
* p*-value for opicapone 50 mg versus levodopa 100 mg	**0.0309**	
OFF-time responder rate (reduction of ≥ 1 h); n/N (%)	69/122 (56.7%)	54/116 (45.6%)
Total ON time (min)		
LS mean (SE) change from baseline	64.2 (10.3)	43.8 (10.6)
LS mean difference versus levodopa 100 mg (95% CI)	20.4 (− 8.8, 49.6)	
* p* value for opicapone 50 mg versus levodopa 100 mg	0.1693	
ON time without dyskinesia (min)		
LS mean (SE) change from baseline	54.9 (12.1)	59.3 (12.4)
LS mean difference versus levodopa 100 mg (95% CI)	− 4.4 (− 38.5, 29.7)	
* p* value for opicapone 50 mg versus levodopa 100 mg	0.8008	
ON time with troublesome dyskinesia (min)		
LS mean (SE) change from baseline	− 3.0 (6.2)	− 2.1 (6.4)
LS mean difference versus levodopa 100 mg (95% CI)	− 0.9 (− 18.3, 16.6)	
* p* value for opicapone 50 mg versus levodopa 100 mg	0.9231	
Asleep (min)		
LS mean (SE) change from baseline	0.9 (6.2)	− 10.1 (6.4)
LS mean difference versus levodopa 100 mg (95% CI)	10.9 (− 6.6, 28.4)	
* p* value for opicapone 50 mg versus levodopa 100 mg	0.2217	
MDS-UPDRS scores		
Part III		
LS mean (SE) change from baseline	− 4.1 (0.6)	− 2.5 (0.6)
LS mean difference versus levodopa 100 mg (95% CI)	− 1.7 (− 3.3, − 0.04)	
* p* value for opicapone 50 mg versus levodopa 100 mg	**0.0445**	
Part IV		
LS mean (SE) change from baseline	− 1.1 (0.2)	− 0.8 (0.2)
LS mean difference versus levodopa 100 mg (95% CI)	− 0.3 (− 0.7, 0.1)	
* p* value for opicapone 50 mg versus levodopa 100 mg	0.1734	
PDQ-8 summary index		
LS mean (SE) change from baseline	− 2.7 (1.0)	− 1.9 (1.0)
LS mean difference versus levodopa 100 mg (95% CI)	− 0.9 (− 3.6, 1.9)	
* p* value for opicapone 50 mg versus levodopa 100 mg	0.5447	
Improvement on CGI-I^a^, n/N (%)	96/114 (84.2%)	84/116 (72.4%)
Improvement on PGI-I^a^, n/N (%)	90/113 (79.7%)	80/116 (69.0%)
Mean (SD) LEDD at end of study	744.4^b^ (218.5)	664.9 (181.7)

Significant *p* values are in bold. ^a^includes any improvement (minimal, much, and very much); ^b^applying 0.5 conversion factor for opicapone (same as tolcapone). Abbreviations: CI, confidence interval; CGI-I, Clinical Global Impressions of Improvement; LS, least square; LEDD, levodopa equivalent daily dose; MDS-UPDRS, Movement Disorder Society-sponsored revision of the Unified Parkinson’s Disease Rating Scale; PDQ, Parkinson’s disease questionnaire; SD, standard deviation; PGI-I, Patient’s Global Impression of Improvement; SE, standard error

Findings from the other diary-reported efficacy endpoints showed non-significant trends in line with the reduction in OFF time. Treatment with opicapone 50 mg resulted in a correspondingly greater increase than treatment with levodopa 100 mg in total ON time (64.2 min vs. 43.8 min; *p* = 0.17); most of which was without *any* dyskinesia. Adjusted mean ON time with troublesome dyskinesia remained unchanged with no difference between groups (–3.0 min with opicapone vs − 2.1 min with levodopa 100 mg; *p* = 0.9231).

**Fig. 2:**
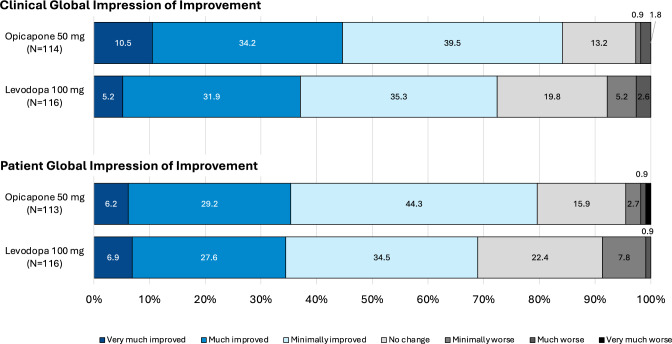
Clinical and patient global impressions of improvement

A higher proportion of patients in the opicapone 50 mg group than those in the levodopa 100 mg group showed improvements (minimally, much, or very much) from baseline to end of study treatment as assessed by CGI-I and PGI-I (Fig. 2). Overall, both groups showed improvements from baseline to end of study in MDS-UPDRS and PDQ-8 scores. The change from baseline to end of study treatment in MDS-UPDRS III (motor) scores (during ON) was significantly greater for the opicapone 50 mg group than the levodopa 100 mg group (− 4.1 vs − 2.5, respectively; *p* = 0.04). While levodopa dose adjustments were minimal during the study, the mean LEDD was higher for the opicapone 50 mg group than for the levodopa 100 mg group (Table 2).

### Safety

Overall, 38% of patients reported at least one AE during treatment with opicapone compared with 25% in the levodopa 100 mg group (Table 3). Dyskinesia was the most frequently reported AE possibly related to the study drug, with the highest incidence observed in the opicapone 50 mg group (9 cases, 7%). The incidence of serious AEs was low (< 3%) and similar between the treatment groups; only one case was judged to be related to the study drug and no deaths occurred during the study.

**Table 3:** Summary of safety (safety analysis set)

	Opicapone 50 mg N = 125	Levodopa 100 mg N = 118
Any AE, n (%) *	47 (37.6)	29 (24.6)
Dyskinesia	9 (7.2)	5 (4.2)
Dizziness	8 (6.4)	3 (2.5)
Nausea	3 (2.4)	4 (3.4)
Insomnia	4 (3.2)	–
Constipation	4 (3.2)	2 (1.7)
Severity, n (%)		
Mild	42 (33.6)	24 (20.3)
Moderate	9 (7.2)	6 (5.1)
Severe	1 (0.8)	1 (0.8)
Serious AEs, n (%)	3 (2.4)	2 (1.7)
Spinal compression fracture	2 (1.6)	–
Subdural hematoma	1 (0.8)	–
Upper limb fracture	–	1 (0.8)
Retinal detachment	–	1 (0.8)
AEs leading to discontinuation, n (%)	4 (3.2)	2 (1.7)
Vertigo	1 (0.8)	–
Somnolence	1 (0.8)	–
Tremor	1 (0.8)	–
Spinal compression fracture	1 (0.8)	–
Upper limb fracture	–	1 (0.8)
Dizziness	1 (0.8)	–
Insomnia	–	1 (0.8)
Rash	1 (0.8)	–
Any drug-related AE, n (%)^a^	32 (25.6)	NA^b^
Dyskinesia	6 (4.8)	–
Dizziness	5 (4.0)	–
Constipation	4 (3.2)	–
Insomnia	4 (3.2)	–
Headache	3 (2.4)	–
Nausea	3 (2.4)	–
Somnolence	2 (1.6)	–
Serious drug-related AE, n (%)	1 (0.8)	–
Subdural hematoma	1 (0.8)	–

*n* represents number of patients

^a^ ≥ 3% of patients; ^b^relationship was only considered for the opicapone group

*AE* adverse event

The percentage of patients who discontinued due to AEs was low (< 5%). There was no common AE leading to discontinuation; all six patients who discontinued due to AEs reported different reasons (Table 3).

## Discussion

Results of this preplanned pooled analysis of two randomized 4-week studies demonstrate that the introduction of adjunct opicapone 50 mg was significantly more efficacious in reducing OFF time compared with adding another levodopa dose of 100 mg in patients with early wearing-off fluctuations. Treatment with opicapone 50 mg was generally safe and well tolerated.

The ADOPTION clinical program was designed to address the common practical question of which strategies should be used to treat early wearing-off symptoms. In this analysis, the mean treatment difference in OFF time between the introduction of adjunct opicapone and the addition of an extra 100 mg/day of levodopa was 29 min. While this is below the minimal clinically important difference (MCID) of 1 h previously reported for OFF time versus placebo [21], it is pertinent that the difference is versus an active intervention (an additional 100 mg of levodopa) and not placebo. Since all patients were already receiving levodopa therapy—and the comparator treatment was an additional levodopa dose—we did not expect any between group differences in motor symptom severity. However, in line with recent data showing that opicapone provides additional symptomatic efficacy in levodopa-treated patients [22], MDS-UPDRS III scores (during ON) showed significantly greater improvements with the introduction of opicapone 50 mg versus the addition of levodopa 100 mg. One of the key findings in the pivotal studies was the maintenance of levodopa dose over an extended period of time (in contrast to the frequent changes often required when trying levodopa modification strategies). Unfortunately, this study was too short to examine this important aspect of therapy, which could also impact the development of dyskinesia.

This pooled analysis was preplanned to reduce recruitment time pressures for each single study, although recruitment was easier in South Korea than Europe (where it was harder to find eligible patients not already on opicapone). When analyzed separately, only the South Korean study showed a significant difference favoring opicapone [15], while OFF time reductions were similar for both treatment arms in the European study (Table e2). However, the European study was smaller and included a subset of ‘outlier’ patients who, in post hoc analyses, showed signs of atypical disease progression. Other potential reasons for the difference between the two studies could include novelty bias (opicapone had only been launched 6 months earlier in South Korea), and in how the additional levodopa doses were given (the method of administering the additional 100 mg levodopa was not specified in the protocol). For example, in the South Korean study, > 90% of patients came into the study on 3 daily intakes of levodopa, and the additional 100 mg was often distributed across those three intakes and not as a 4th full intake. By contrast, about half of the patients in the European study were on 4 daily levodopa doses, and the additional 100 mg of levodopa was frequently added as a full 4th or 5th intake (i.e., shortening of the interdose interval). The relative efficacy of increasing levodopa dose frequency (fractionation) vs increasing individual doses has not been well studied.

Opicapone was safe and well tolerated, and most AEs, in both groups, were as expected for a dopaminergic replacement therapy. Consistent with the higher LEDD in the opicapone group, the incidence of treatment-emergent dyskinesia reported as an AE was slightly higher in the opicapone than the levodopa 100 mg group; however, this was mainly reported as mild. On the other hand, patients showed an increase in ON time without any dyskinesia. Recent pharmacokinetic data suggest that this may be because opicapone acts to improve the levodopa AUC *without* significant impact the levodopa C_max_ [23].

Strengths of the ADOPTION clinical program lie in the similar designs of the study which enabled patient level integration of data. Similar studies in other countries are currently under consideration and could be added to the dataset. Although open-label, the program was designed to include two randomized studies that evaluated the introduction of adjunct opicapone against one of the most commonly used and potent strategies—namely levodopa dose increase. The study also specifically recruited patients who were earlier in their disease course than the pivotal studies (patients in the BIPARK studies had more advanced PD as evidence by a longer disease duration of ~ 8 years; baseline daily OFF time of ~ 6 h; and a daily levodopa dose of ~ 700 mg/day); as such, the results of the current study should not be compared to the pivotal studies. Limitations of the program lie in its open-label design, variability in the ways that the additional 100 mg levodopa was given (the manner was not prespecified nor recorded, although the European preference for giving the 100 mg levodopa as a 4th or 5th intake vs the South Korean preference for remaining at 3 intakes is evidenced by diary data) and short study duration. Further prospective studies powered to evaluate direct comparisons between opicapone and different levodopa regimens are warranted. Previous post hoc analyses of the pivotal studies have suggested added benefit of opicapone as a first-line adjunctive therapy to levodopa [14]. While patient numbers were too low in the present analysis, it would also be of interest to compare outcomes in patients previously on levodopa monotherapy (i.e., opicapone as first-line adjunct therapy).

In summary, the results of this pooled analysis indicate that opicapone is a well-tolerated and effective option for patients who have developed the early signs of wearing-off.

## Supplementary Information

Below is the link to the electronic supplementary material.

Supplementary file1 (DOCX 40 kb)

## Data Availability

The data that support the findings of this study are available from the corresponding author upon reasonable request.
