# Correction: Opicapone for the treatment of early wearing-off in levodopa-treated Parkinson’s disease: pooled analysis of patient level data from two randomized open-label studies

**DOI:** 10.1007/s00415-025-13122-z

**Published:** 2025-08-01

**Authors:** Joaquim J. Ferreira, Jee-Young Lee, Hyeo-il Ma, Beomseok Jeon, Werner Poewe, Angelo Antonini, Fabrizio Stocchi, Daniela M. Rodrigues, Miguel M. Fonseca, Guillermo Castilla-Fernández, Joerg Holenz, José-Francisco Rocha, Olivier Rascol, on behalf of the ADOPTION study investigators

**Affiliations:** 1 IMM - Instituto de Medicina Molecular João Lobo Antunes, Faculdade de Medicina, Universidade de Lisboa, Lisbon, Portugal; 2 CNS – Campus Neurológico, Torres Vedras, Portugal; 3 Department of Neurology, SMG-SNU Boramae Medical Center, Seoul, Korea; 4 Department of Neurology, Hallym University Sacred Heart Hospital, Anyang, Korea; 5 Department of Neurology, Seoul National University Hospital, Seoul, Korea; 6 Department of Neurology, Medical University of Innsbruck, Innsbruck, Austria; 7 Department of Neurosciences, University of Padova, Padua, Italy; 8 IRCCS San Raffaele Roma, Rome, Italy; 9 San Raffaele University, Rome, Italy; 10 BIAL – Portela and Ca S.A., Coronado, Portugal; 11 BIAL - R&D Investments, S.A., Coronado, Portugal; 12 Clinical Investigation Center CIC1436, Departments of Neurosciences and Clinical Pharmacology and NS-Park/FCRIN Network, University of Toulouse 3, University Hospital of Toulouse, INSERM, Toulouse, France

**Correction: Journal of Neurology (2024) 271:6729–6738** 10.1007/s00415-024-12614-8

In the original version of this article, the affiliation details for author Fabrizio Stocchi were incorrectly given as

University San Raffaele Roma and Institute for Research and Medical Care IRCCS San Raffaele, Rome, Italy

but should have been

IRCCS San Raffaele Roma, Rome, Italy

San Raffaele University, Rome, Italy

The funding information section was incorrectly given as

Open access funding provided by FCT|FCCN (b-on). Joaquim J. Ferreira has provided consultancy and received speaker fees from Lundbeck, BIAL, Biogen, Acadia, Abbvie, Sunovion Pharmaceuticals, Zambon, Affiris, Roche, ONO and SK Chemicals and has received grants from Abbvie, Bial, Medtronic and Angelini. Jee-Young Lee received a research grant of NRF funded by the MSIT of Korea and a multidisciplinary research grant-in-aid and a focused clinical research grant-in-aid from the Seoul Metropolitan Government-Seoul National University (SMG-SNU) Boramae Medical Center, and speaker honorarium from Esai Korea, Bial, SK chemicals and Scientific Advisory Board of Regeners Inc. Beomseok Jeon has received research grant from Peptron and Abbvie Korea and has participated in an expert panel for Aspen Neuroscience. Werner Poewe has received lecture fees and honoraria for consultancy in relation to clinical drug development programs from Alterity, AbbVie, Affiris, AstraZeneca, Axovant, BIAL, Biogen, Britannia, Lilly, Lundbeck, NeuroDerm, Neurocrine, Denali Pharmaceuticals, Orion Pharma, Roche, Stada, Sunovion, Takeda, UCB and Zambon, as well as grant support from the MJFF and the EU FP7 & Horizon 2020 programs. Angelo Antonini has received compensation for consultancy and speaker-related activities from UCB, Boehringer Ingelheim, Britannia, AbbVie, Zambon, Bial, NeuroDerm, Theravance Biopharma, Roche; he receives research support from Chiesi Pharmaceuticals, Lundbeck, Horizon 2020—Grant 825785, Horizon2020 Grant 101016902, Ministry of Education University and Research (MIUR) Grant ARS01_01081, Cariparo Foundation. He serves as consultant for Boehringer Ingelheim for legal cases on pathological gambling; owns Patent WO2015110261-A1; and owns shares in PD Neurotechnology Limited. Fabrizio Stocchi has received compensation for consultancy and speaker-related activities from Lundbeck, UCB, Chiesi, Zambon, Britannia, Cynapsus, Sunovion, Kyowa, Abbvie, NeuroDerm, Biogen, Bial. Daniela M. Rodrigues, Miguel M. Fonseca, Guillermo Castilla-Fernández, Joerg Holenz and José-Francisco Rocha are employed by BIAL. Olivier Rascol has participated in advisory boards and/or provided consultancy for AbbVie, Adamas, Acorda, Addex, AlzProtect, ApoPharma, AstraZeneca, Axovant, Bial, Biogen, Britannia, Buckwang, CereSpir, Clevexel, Denali, INC Research, IPMDS, Lundbeck, Lupin, Merck, MundiPharma, NeurATRIS, NeuroDerm, Novartis, ONO Pharma, Osmotica, Parexel, Pfizer, Prexton Therapeutics, Quintiles, Roche, Sanofi, Servier, Sunovion, Theranexus, Takeda, Teva, UCB, Vectura, Watermark Research, XenoPort, XO, Zambon; received grants from Agence Nationale de la Recherche (ANR), CHU de Toulouse, France-Parkinson, INSERMDHOS Recherche Clinique Translationnelle, MJ Fox Foundation, Programme Hospitalier de Recherche Clinique, European Commission (FP7, H2020), Cure Parkinson UK; and received a grant to participate in a symposium and contribute to the review of an article by the International Parkinson and Movement Disorder Society.

and should have read

Open access funding provided by FCT|FCCN (b-on). Joaquim J. Ferreira has provided consultancy and received speaker fees from Lundbeck, BIAL, Biogen, Acadia, Abbvie, Sunovion Pharmaceuticals, Zambon, Affiris, Roche, ONO and SK Chemicals and has received grants from Abbvie, Bial, Medtronic and Angelini. Jee-Young Lee received a research grant of NRF funded by the MSIT of Korea and a multidisciplinary research grant-in-aid and a focused clinical research grant-in-aid from the Seoul Metropolitan Government-Seoul National University (SMG-SNU) Boramae Medical Center, and speaker honorarium from Esai Korea, Bial, SK chemicals and Scientific Advisory Board of Regeners Inc. Beomseok Jeon has received research grant from Peptron and Abbvie Korea and has participated in an expert panel for Aspen Neuroscience. Werner Poewe has received lecture fees and honoraria for consultancy in relation to clinical drug development programs from Alterity, AbbVie, Affiris, AstraZeneca, Axovant, BIAL, Biogen, Britannia, Lilly, Lundbeck, NeuroDerm, Neurocrine, Denali Pharmaceuticals, Orion Pharma, Roche, Stada, Sunovion, Takeda, UCB and Zambon, as well as grant support from the MJFF and the EU FP7 & Horizon 2020 programs. Angelo Antonini has received compensation for consultancy and speaker-related activities from UCB, Boehringer Ingelheim, Britannia, AbbVie, Zambon, Bial, NeuroDerm, Theravance Biopharma, Roche; he receives research support from Chiesi Pharmaceuticals, Lundbeck, Horizon 2020—Grant 825785, Horizon2020 Grant 101016902, Ministry of Education University and Research (MIUR) Grant ARS01_01081, Cariparo Foundation. He serves as consultant for Boehringer Ingelheim for legal cases on pathological gambling; owns Patent WO2015110261-A1; and owns shares in PD Neurotechnology Limited. Fabrizio Stocchi has received compensation for consultancy and speaker-related activities from Lundbeck, UCB, Chiesi, Zambon, Britannia, Cynapsus, Sunovion, Kyowa, Abbvie, NeuroDerm, Biogen, Bial. Fabizio Stocchi work is supported by funding of the Italian Ministry of Health [Ricerca Corrente]." Daniela M. Rodrigues, Miguel M. Fonseca, Guillermo Castilla-Fernández, Joerg Holenz and José-Francisco Rocha are employed by BIAL. Olivier Rascol has participated in advisory boards and/or provided consultancy for AbbVie, Adamas, Acorda, Addex, AlzProtect, ApoPharma, AstraZeneca, Axovant, Bial, Biogen, Britannia, Buckwang, CereSpir, Clevexel, Denali, INC Research, IPMDS, Lundbeck, Lupin, Merck, MundiPharma, NeurATRIS, NeuroDerm, Novartis, ONO Pharma, Osmotica, Parexel, Pfizer, Prexton Therapeutics, Quintiles, Roche, Sanofi, Servier, Sunovion, Theranexus, Takeda, Teva, UCB, Vectura, Watermark Research, XenoPort, XO, Zambon; received grants from Agence Nationale de la Recherche (ANR), CHU de Toulouse, France-Parkinson, INSERMDHOS Recherche Clinique Translationnelle, MJ Fox Foundation, Programme Hospitalier de Recherche Clinique, European Commission (FP7, H2020), Cure Parkinson UK; and received a grant to participate in a symposium and contribute to the review of an article by the International Parkinson and Movement Disorder Society.

