# Supplementary material for: Opicapone for the treatment of early wearing-off in levodopa-treated Parkinson’s disease: pooled analysis of patient level data from two randomized open-label studies
Source: J Neurol. 2024 Aug 21;271(10):6729–38. doi: 10.1007/s00415-024-12614-8 (PMC11447072; doi:10.1007/s00415-024-12614-8)
Supplement: Supplementary file 1 — Supplementary file1 (DOCX 40 kb) [file 415_2024_12614_MOESM1_ESM.docx]

# SUPPLEMENTARY INFORMATION

**Figure S1. Patient disposition.** L-DOPA, levodopa; FAS, Full Analysis Set


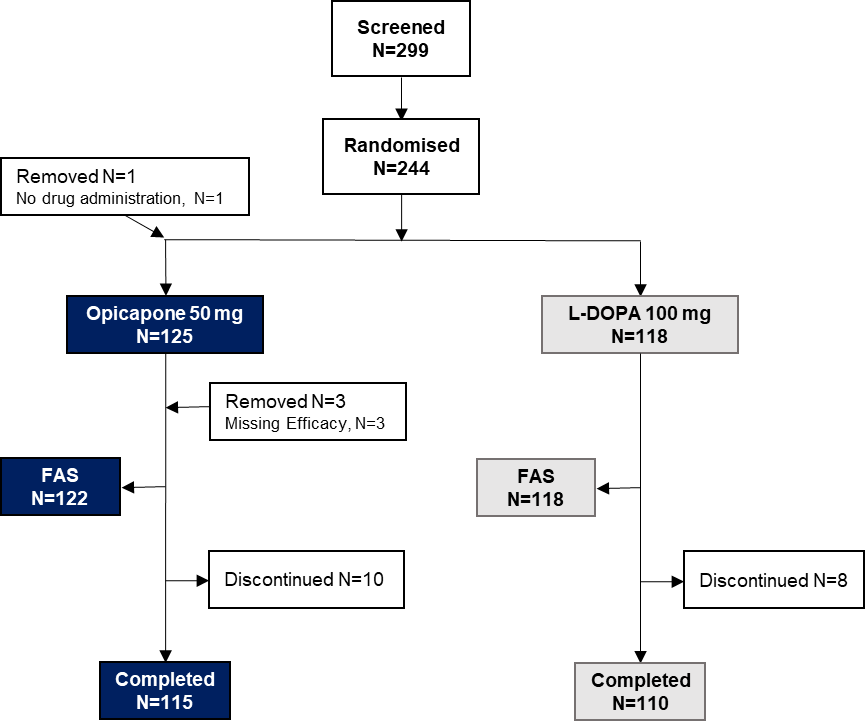


**Table e1. Baseline characteristics per study**

|  | **South Korea^1^**  **N=169** | **Europe**  **N=75** |
| --- | --- | --- |
| Age, year | 64.1 (7.7) | 64.8 (10.7) |
| Male, n (%) | 83 (49.1) | 45 (60.0) |
| Hoehn & Yahr, stage | 2.0 (0.5) | 2.0 (0.4) |
| PD duration, year | 5.3 (3.6) | 4.9 (3.5) |
| MDS-UPDRS motor score | 22.8 (10.8) | 27.1 (11.6) |
| Daily OFF time, hours | 3.4 (1.0) | 3.5 (1.0) |
| Total ON time, hours | 13.0 (1.6) | 12.2 (1.5) |
| ON time without dyskinesia, hours | 11.3 (3.2) | 11.5 (2.5) |
| Levodopa dose at baseline, mg | 408.3 (123.9) | 398.5 (106.5) |
| Patients receiving 3 or 4 levodopa intakes per day, n (%)  *3 intakes*  *4 intakes* | 155 (91.7)  14 (8.3) | 38 (50.7)  36 (48.0) |
| **Concomitant therapy, n (%)^a^**  **Dopamine agonist**  Pramipexole  Rotigotine  Ropinirole  **MAO-B inhibitor**  Rasagiline  Safinamide  Selegiline | 144 (85.2)  115 (68.0)  90 (62.5)  -  33 (22.9)  89 (52.7)  84 (58.3)  4 (2.8)  2 (1.4) | 61 (81.3)  37 (49.2)  19 (25.3)  10 (13.3)  8 (10.6)  45 (60.0)  26 (34.7)  18 (24.0)  1 (1.3) |
| LEDD^a^, mg | 580.8 (179.8) | 542.2 (156.2) |

*LEDD, levodopa equivalent daily dose; MAO-B, monoamine oxidase B; MDS, Movement Disorder Society; UDPRS, Unified Parkinson’s Disease Rating Scale; PD, Parkinson’s disease; ^a^calculated for Levodopa, MAO-B inhibitors and dopamine agonists only*

**Table e2. Change in OFF time in subgroups (minutes)**

| **Opicapone 50mg arm** | LS mean (SE) change from baseline |
| --- | --- |
| South Korean analysis^1^ (n=84)  European analysis (n=38)  Males from integrated analysis* (n=65)  Females from integrated analysis* (n=57) | –62.1 (9.8)  –64.4 (17.6)  –69.8 (12.4)  –54.8 (12.5) |
| **Levodopa 100 mg arm** | LS mean (SE) change from baseline |
| South Korean analysis^1^ (n=81)  European analysis (n=37)  Males from integrated analysis* (n=63)  Females from integrated analysis* (n=55) | -16.7 (10.0)  -73.0 (18.3)  -39.9 (12.8)  -27.0 (12.7) |

*Integrated analysis from two similarly designed 4-week, open-label studies conducted in South Korea (NCT04821687) and Europe (NCT04990284).

**Reference**

1. Lee JY, Ma HI, Ferreira JJ, Rocha JF, Sung YH, Song IU, Ahn TB, Kwon DY, Cheon SM, Kim JM, Lee CS, Lee PH, Park JH, Lee JH, Park MY, Kim SJ, Baik JS, Choi SM, Shin HW, Lee HW, Kang SY, Jeon B (2024) Opicapone to Treat Early Wearing-off in Parkinson's Disease Patients: The Korean ADOPTION Trial. Mov Disord Clin Pract 11:655-665.
